# Host-associated *Enterococcus faecium* isolated from Naleh fish (*Barbonymus* sp.) enhances growth performance, feed utilization, and water quality in common carp (*Cyprinus carpio*)

**DOI:** 10.14202/vetworld.2026.2585-2596

**Published:** 2026-06-25

**Authors:** Cut Yulvizar, Yulia Sari Ismail, Cut Nanda Defira, Puja Nabila Agustina, Chendrastyan Meuraxa, Dewi Yunita, Zainal A Muchlisin, Suhartono Suhartono

**Affiliations:** 1 Graduate School of Mathematics and Applied Science, Universitas Syiah Kuala, Banda Aceh, 23111, Indonesia; 2 Department of Biology, Faculty of Mathematics and Natural Sciences, Universitas Syiah Kuala, Banda Aceh, 23111, Indonesia; 3 Department of Agricultural Product Technology, Faculty of Agriculture, Universitas Syiah Kuala, Banda Aceh, 23111, Indonesia; 4 Department of Marine Sciences, Faculty of Marine and Fisheries, Universitas Syiah Kuala, Banda Aceh, 23111, Indonesia

**Keywords:** aquaculture, common carp, *Enterococcus faecium*, feed efficiency, growth performance, host-associated probiotic, lactic acid bacteria, water quality

## Abstract

**Background and Aim:**

Common carp (*Cyprinus carpio*) is an economically important freshwater aquaculture species; however, its production is often constrained by poor feed efficiency (FE), disease susceptibility, and deteriorating water quality. Probiotics, particularly lactic acid bacteria (LAB), have been widely applied to improve fish performance and culture conditions. Host-associated probiotics candidate isolated from phylogenetically related fish species may provide superior colonization and biological benefits compared with non-native strains. Indigenous LAB isolated from Naleh fish (*Barbonymus* sp.), a native freshwater species in western Aceh, Indonesia, may serve as a host-associated probiotic candidate for common carp (*C. carpio*), as both species belong to the family Cyprinidae. This study evaluated the effects of LAB NJ19, an indigenous isolate obtained from the digestive tract of Naleh fish (*Barbonymus* sp.), on growth performance, feed utilization, survival, and water quality in common carp.

**Materials and Methods:**

A 40-day feeding trial was conducted using a completely randomized design with three treatments and four replicates: control diet without probiotics, diet supplemented with LAB NJ19 at 1 g/100 g feed, and diet supplemented with the commercial probiotic EM-4 (PT Songgolangit Persada, Jakarta, Indonesia) at 1 g/100 g feed. Juvenile *C. carpio* with an initial weight of 1.14 ± 0.07 g and length of 4.15 ± 0.10 cm were stocked at 10 fish per container. Molecular identification of LAB NJ19 was performed through *16S rRNA* gene sequencing. Growth performance, feed conversion ratio (FCR), FE, survival rate (SR), and water quality parameters were evaluated. Data were analyzed using one-way analysis of variance followed by Duncan’s multiple-range test, with significance set at p < 0.05.

**Results:**

Sequence analysis demonstrated that LAB NJ19 shared 99.46% similarity with *Enterococcus faecium* and clustered within the *E. faecium* clade. Dietary supplementation with probiotics significantly improved growth performance and feed utilization compared with the control (p < 0.05). Fish fed LAB NJ19 exhibited the highest absolute weight gain (7.31 ± 0.29 g), specific growth rate (2.13 ± 0.03%/day), and FE (81.22 ± 3.26%), together with the lowest FCR (1.23 ± 0.04). The commercial probiotic EM-4 also enhanced growth and feed utilization, but was less effective than LAB NJ19 in improving growth-related parameters. SRs ranged from 67.50% to 77.50% and did not differ significantly among treatments (p > 0.05). Water quality remained within acceptable limits throughout the experiment.

**Conclusion:**

The indigenous host-associated probiotic candidate LAB NJ19, identified as *E. faecium*, significantly enhanced growth performance, feed utilization efficiency, and water quality in *C. carpio*. These findings highlight the potential of host-associated LAB as sustainable alternatives to commercial probiotics and support their use to improve productivity and environmental management in freshwater aquaculture systems.

## INTRODUCTION

Common carp (*Cyprinus carpio*) is a major freshwater aquaculture commodity due to its high environmental adaptability, prolific spawning capacity, and rapid growth rate [1–3]. However, common carp production continues to face several constraints, including poor feed efficiency (FE), disease outbreaks, and deteriorating water quality [4, 5]. Suboptimal feed quality adversely affects fish growth, survival rate (SR), and FE [6, 7]. Feed quality is therefore a critical determinant of aquaculture success, and the application of probiotics, particularly lactic acid bacteria (LAB), has emerged as a promising strategy to address these challenges [8, 9]. LAB have been extensively investigated as probiotic candidates in aquaculture because they naturally inhabit fish digestive tracts and have demonstrated efficacy in promoting growth and maintaining host health [10, 11].

Probiotics are defined as live microorganisms that, when administered in adequate amounts, confer beneficial effects on the host by improving nutrient digestibility, preventing disease, and maintaining favorable water quality conditions [12, 13]. LAB produce lactic acid during carbohydrate metabolism and synthesize antimicrobial compounds such as organic acids, hydrogen peroxide, carbon dioxide, and bacteriocins that inhibit the proliferation of pathogenic microorganisms [14, 15]. Consequently, dietary supplementation with probiotic bacteria can improve nutrient absorption efficiency and promote fish growth [16, 17]. Several probiotic bacteria have been isolated from aquatic organisms, including *Micrococcus*, *Staphylococcus*, and *Bacillus* species from mackerel (*Rastrelliger* sp.) [18]; *Lactobacillus acidophilus* and *Micrococcus lylae* from milkfish (*Chanos chanos*) [19]; *Lysinibacillus* sp. and *Citrobacter freundii* from Nile tilapia (*Oreochromis niloticus*) [20]; *Enterococcus faecium* from the same species [21]; and *Vibrio* sp., *Staphylococcus* sp., and *Pseudoalteromonas* sp. from whiteleg shrimp (*Litopenaeus vannamei*) [22]. Therefore, the use of LAB as probiotics represents a promising approach to improving gut health, nutrient absorption, and overall fish performance in aquaculture systems.

The use of indigenous microbial species as probiotics is considered advantageous because these microorganisms originate from environments and hosts with physiological characteristics similar to those of the target species [23]. This advantage is associated with the influence of feeding habits, ecological niches, and trophic levels on the composition of intestinal microbiota, which may enhance probiotic colonization and increase beneficial effects in the host [24]. Probiotics derived from closely related host species have been reported to exhibit superior colonization ability and biological functionality because their adaptation to host physiological conditions facilitates successful establishment within the gastrointestinal tract [25, 26]. Therefore, host-associated probiotics are increasingly regarded as promising alternatives to non-native commercial probiotic strains in aquaculture.

Despite extensive research on probiotic applications in cyprinid fish, information regarding LAB isolated from Naleh fish (*Barbonymus* sp.) as potential probiotics for common carp remains unavailable. Naleh fish (*Barbonymus* sp.) is an endemic freshwater species found in western Aceh, Indonesia, particularly in the rivers of Nagan Raya Regency [27, 28]. This species belongs to the family Cyprinidae and order Cypriniformes, making it phylogenetically related to *C. carpio* [27]. Naleh fish exhibits omnivorous feeding behavior with herbivorous tendencies and inhabits freshwater ecosystems that share ecological characteristics with those used for common carp culture [28].

Previous studies demonstrated that LAB NJ19, isolated from the digestive tract of Naleh fish, possesses promising probiotic properties. LAB NJ19 exhibited inhibitory activity against *Aeromonas hydrophila*, a pathogen that causes Motile Aeromonas septicemia (MAS) and substantial mortality in cultured fish. In addition, LAB NJ19 showed high amylase activity, suggesting a strong capacity to improve carbohydrate digestion and nutrient utilization efficiency. These characteristics indicate that LAB NJ19 has the potential to serve as a host-associated probiotic that enhances digestive processes, nutrient assimilation, and overall fish performance. Based on the taxonomic relationship between Naleh fish and common carp and the ecological adaptation of LAB NJ19 to the digestive tract of a closely related host species, this isolate may provide superior colonization and probiotic functionality compared with conventional commercial probiotics.

Although previous investigations have characterized the antimicrobial and enzymatic properties of LAB NJ19, no studies have evaluated its efficacy as a dietary probiotic in common carp culture systems. Furthermore, the potential advantages of using a probiotic isolated from a phylogenetically related host species have not been investigated under practical aquaculture conditions. Consequently, there is limited information regarding whether host-associated LAB derived from *Barbonymus* sp. can improve growth performance, FE, SR, and water quality in common carp culture. Addressing this knowledge gap is important because host-associated probiotics may provide superior colonization, improved digestive performance, enhanced nutrient utilization, and better environmental benefits than conventional commercial probiotics. Therefore, scientific evidence is needed to validate the effectiveness of LAB NJ19 as a sustainable probiotic candidate for freshwater aquaculture and to determine whether host-associated probiotics can provide measurable advantages over commercially available probiotic products.

Therefore, this study aimed to evaluate the effects of dietary supplementation with LAB NJ19, an indigenous probiotic isolated from the digestive tract of Naleh fish (*Barbonymus* sp.), on growth performance, FE, SR, and water quality in common carp (*C. carpio*). In addition, the effectiveness of LAB NJ19 was compared with that of a commercially available probiotic to determine its potential as a sustainable host-associated probiotic candidate for freshwater aquaculture applications.

## MATERIALS AND METHODS

### Ethical approval

This study was reviewed and approved by the Animal Ethics Committee of Universitas Syiah Kuala, Banda Aceh, Indonesia (Approval No. 457/KEPH/I/2024; dated January 17, 2024). All procedures involving *C. carpio* were conducted in accordance with institutional and internationally accepted guidelines for the ethical care and use of animals in research. Fish were acclimatized before the experiment and maintained under controlled husbandry conditions with continuous aeration, regular water exchange, and daily monitoring of water quality, behavior, feeding activity, morbidity, and mortality. Handling and sampling procedures were performed carefully to minimize stress and unnecessary suffering. No antibiotics or therapeutic agents were administered during the feeding trial, and no severe morbidity or welfare condition requiring euthanasia was observed. The study used only juvenile fish to evaluate dietary probiotic supplementation and growth performance, and all efforts were made to ensure animal welfare throughout the 40-day experimental period.

### Study period and location

This study was conducted from February to September 2025 and consisted of two sequential phases, namely *in vitro* and *in vivo* investigations. The *in vitro* phase included probiotic regeneration, culture preparation, harvesting, and probiotic feed formulation. It was carried out at the Microbiology Laboratory, Department of Biology, Faculty of Mathematics and Natural Sciences, Universitas Syiah Kuala, Banda Aceh, Indonesia. The *in vivo* phase involved evaluating the effects of probiotic supplementation on common carp growth performance and was conducted at the Fish Hatchery and Breeding Laboratory, Faculty of Marine and Fisheries, Universitas Syiah Kuala, Banda Aceh, Indonesia. The *in vitro* phase was completed before initiation of the *in vivo* feeding trial.

### Study design

The experiment was conducted using a completely randomized design with three dietary treatments and four replicates per treatment. The experimental factor evaluated was probiotic supplementation of common carp feed. The treatments consisted of a control diet without probiotic supplementation (P1), a diet supplemented with the LAB NJ19 at 1 g/100 g feed (P2), and a diet supplemented with the commercial probiotic EM-4 (PT Songgolangit Persada, Jakarta, Indonesia) at 1 g/100 g feed (P3). The probiotic inclusion level of 1% was selected based on previous studies reporting beneficial effects of similar supplementation rates on fish growth performance and feed utilization in aquaculture systems [29]. Twelve experimental units consisting of 40-L containers were randomly assigned to the three treatments.

### Container preparation

A 1000-L container containing water at a depth of approximately 70 cm was used during the acclimation period, whereas twelve 40-L containers were prepared for the feeding trial. All containers were thoroughly cleaned with soap, rinsed, dried, and exposed to direct sunlight for 3 h to minimize microbial contamination and prevent mold growth. The prepared containers were filled with 20 L of dechlorinated freshwater and continuously aerated for 24 h before fish stocking. Aeration was maintained throughout the experimental period using air pumps and air stones.

### Test fish preparation

Common carp juveniles measuring 3–4 cm in length were acclimated for 2 days before the experiment. Fish with an initial average body weight of 1.14 ± 0.07 g and an average length of 4.15 ± 0.10 cm were used. Fish were stocked at a density of 10 fish per container containing 20 L of water and maintained for 40 days.

### Identification of probiotic LAB NJ19

The probiotic LAB NJ19 used in this study was previously isolated from the digestive tract of Naleh fish (*Barbonymus* sp.) and maintained at the Microbiology Laboratory, Faculty of Mathematics and Natural Sciences, Universitas Syiah Kuala. Preliminary identification of the isolate was conducted based on morphological and biochemical characteristics, including colony morphology on de Man, Rogosa, and Sharpe agar, Gram staining, and catalase testing. The isolate was Gram-positive and catalase-negative, which are typical features of LAB.

For molecular identification, genomic DNA was extracted from LAB NJ19, and the *16S rRNA* gene was amplified using universal *16S rRNA* gene primers. The amplified product was sequenced by Macrogen (Seoul, South Korea). The obtained sequence was compared with sequences available in the National Center for Biotechnology Information database using the Basic Local Alignment Search Tool to determine the phylogenetic affiliation of the isolate.

### Test feed preparation

LAB NJ19 is an indigenous LAB isolate obtained from the digestive tract of Naleh fish and maintained in the Microbiology Laboratory, Faculty of Mathematics and Natural Sciences, Universitas Syiah Kuala. This isolate exhibits moderate antibacterial activity against *A. hydrophila*.

For feed preparation, LAB NJ19 was cultured on de Man, Rogosa, and Sharpe agar medium to obtain a bacterial density of approximately 10^8^ colony-forming units/mL. The bacterial cells were harvested by centrifugation at 5,000 × *g* for 30 min and subsequently resuspended in phosphate-buffered saline. A 1-mL aliquot of the bacterial suspension was mixed with 100 g of commercial feed, and 2% egg white was added as a binder. The coated feed was air-dried at room temperature, prepared every 10 days to maintain probiotic viability, and stored at 4°C until use [29].

The bacterial suspension used for feed supplementation had an initial density of approximately 10^8^ colony-forming units/mL. Although the exact viable count in the final feed was not determined, the preparation procedure was applied consistently across all treatments to ensure comparable probiotic exposure.

The commercial feed used in this study was PF-1000 (MS Prima Feed, Surabaya, Indonesia) with a pellet size of 1.3–1.7 mm. According to the manufacturer’s specifications, the feed contained approximately 39%–41% crude protein, ≥5% crude lipid, ≤6% crude fiber, ≤16% ash, and ≤10% moisture.

### Fish rearing and feeding

**Figure 1:**
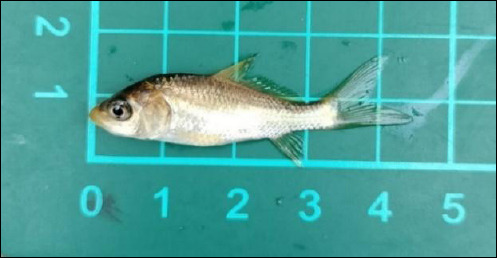
Representative common carp (*Cyprinus carpio*) juveniles (3–5 cm total length) maintained in 40-L containers at a stocking density of 10 fish per container during the 40-day probiotic feeding trial.

Fish with an initial average body weight of 1.14 ± 0.07 g and length of 4.15 ± 0.10 cm were reared for 40 days (Figure 1). Water was siphoned every 2 days to remove accumulated debris and reduce ammonia buildup, and clean freshwater was then added to restore the original volume.

Fish were fed three times daily. Morning feeding consisted of unsupplemented feed, whereas probiotic-supplemented feed was provided during midday and evening feedings. Feeding was performed *ad libitum* until fish ceased feeding activity. Feed consumption was recorded daily to estimate feed intake and support the calculation of the feed conversion ratio (FCR). No antibiotics or other therapeutic agents were administered during the experimental period.

### Study parameters

Growth performance and feed utilization were evaluated using the following formulas:

SGR = [(ln Wt − ln W_0_)/T] × 100

where SGR is the specific growth rate (%/day), W_0_ is the average fish weight at the beginning of the study (g), Wt is the average fish weight at time t (g), and T is the rearing period (days).

W = Wt − W_0_

where W is the absolute weight gain (g), Wt is the final individual fish weight (g/fish), and W_0_ is the initial individual fish weight (g/fish).

L = Lt − L_0_

where L is the absolute length gain (cm), Lt is the final fish length (cm), and L_0_ is the initial fish length (cm).

FCR = F/(Wt − W_0_)

where FCR is the feed conversion ratio, F is the amount of feed consumed (g), Wt is the final average fish weight (g), and W_0_ is the initial average fish weight (g).

FE = (1/FCR) × 100

where FE is feed efficiency (%) and FCR is the feed conversion ratio.

SR (%) = (Nt/N_0_) × 100

where SR is the survival rate, Nt is the number of fish at the end of the study, and N_0_ is the number of fish at the beginning of the study.

### Water quality parameters

Water quality parameters included temperature, dissolved oxygen (DO), and pH. Measurements were performed daily using standard water quality instruments. Approximately 50% of the water volume was replaced every 2 days to maintain suitable culture conditions.

### Statistical analysis

Data are presented as mean ± standard deviation. Before analysis, data were tested for normality using the Shapiro–Wilk test and for homogeneity of variance using Levene’s test. Differences among treatments were evaluated using one-way analysis of variance. When significant differences were detected (p < 0.05), Duncan’s multiple-range test was used for post hoc comparisons. FCR was calculated using recorded feed consumption and weight gain data collected during the experimental period. Statistical analyses were performed using SPSS version 25.

## RESULTS

### Identification and characterization of LAB NJ19

**Figure 2:**
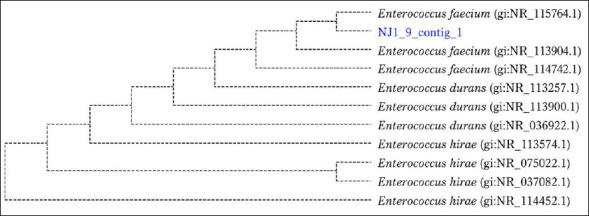
Phylogenetic tree based on *16S rRNA* gene sequences showing the clustering of lactic acid bacteria isolate NJ19 within the *E. faecium* clade, confirming its taxonomic identity.

**Figure 3:**
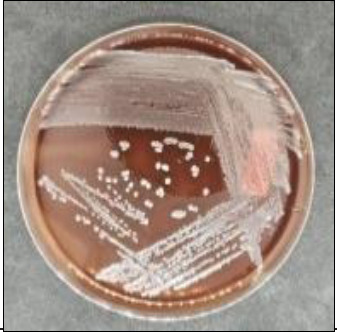
Colony morphology of lactic acid bacteria isolate NJ19 cultured on de Man, Rogosa, and Sharpe agar, showing characteristic white, convex colonies with smooth margins.

**Figure 4:**
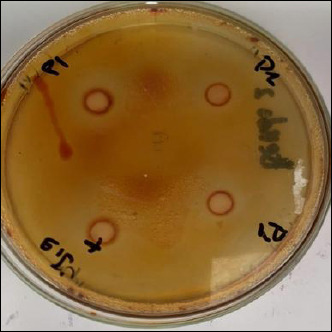
Amylase activity of probiotic lactic acid bacteria isolate NJ19 is indicated by clear-zone formation on starch agar.

LAB NJ19 was successfully identified through *16S rRNA* gene sequencing. Sequence analysis revealed that LAB NJ19 shared 99.46% similarity with *E. faecium*, indicating reliable species-level identification (Figure 2). Morphological characterization showed that LAB NJ19 formed colonies with smooth margins, white pigmentation, convex elevation, circular shape, and coccus-shaped cells (Figure 3). LAB NJ19 was cultured on de Man, Rogosa, and Sharpe agar, which supported optimal growth of the isolate. Qualitative screening demonstrated that LAB NJ19 produced the largest clear zone among all LAB isolates, indicating high amylase activity (Figure 4) and also exhibited potential protease and lipase activities. Common carp (*C. carpio*) and Naleh fish (*Barbonymus* sp.) both belong to the family Cyprinidae. Because LAB NJ19 was isolated from the digestive tract of Naleh fish, it may serve as a host-associated probiotic candidate for common carp culture.

### Effects of LAB NJ19 on growth performance and feed utilization

The administration of LAB NJ19 and the commercial probiotic EM-4 significantly affected growth performance and FE (p < 0.05), whereas SR was not significantly influenced by dietary treatment (p > 0.05) (Table 1).

**Table 1:** Growth performance, feed utilization efficiency, and survival rate of common carp (*C. carpio*) fed diets supplemented with indigenous lactic acid bacteria NJ19 or commercial probiotic EM-4 for 40 days.

Treatment	Absolute weight gain (g)	Absolute length gain (cm)	SGR (%/day)	FCR	FE (%)	SR (%)
P1 (Control)	6.26 ± 0.18ᵃ	3.43 ± 0.25ᵃ	1.99 ± 0.01ᵃ	1.48 ± 0.12ᵇ	67.94 ± 5.34ᵃ	67.50 ± 9.57ᵃ
P2 (LAB NJ19)	7.31 ± 0.29ᶜ	3.95 ± 0.26ᵇ	2.13 ± 0.03ᶜ	1.23 ± 0.04ᵃ	81.22 ± 3.26ᵇ	77.50 ± 12.58ᵃ
P3 (Commercial probiotic EM-4)	6.88 ± 0.12ᵇ	3.95 ± 0.22ᵇ	2.07 ± 0.01ᵇ	1.30 ± 0.02ᵃ	76.44 ± 1.42ᵇ	72.50 ± 9.57ᵃ

Values are presented as mean ± standard deviation. Different superscript letters within a column indicate significant differences among treatments (p < 0.05). FCR = Feed conversion ratio, FE = Feed efficiency, SGR = Specific growth rate, SR = Survival rate.

Fish fed LAB NJ19 (P2) exhibited superior growth performance compared with fish receiving the control diet (P1) or the commercial probiotic treatment (P3). The average absolute weight gain in P2 reached 7.31 ± 0.29 g, which was significantly higher than that observed in P3 (6.88 ± 0.12 g) and P1 (6.26 ± 0.18 g).

A similar trend was observed for absolute length gain. Fish in P2 achieved a length gain of 3.95 ± 0.26 cm, which was significantly higher than that of P1 (3.43 ± 0.25 cm) but not significantly different from that of P3 (3.95 ± 0.22 cm).

The highest SGR was recorded in P2 (2.13 ± 0.03%/day), followed by P3 (2.07 ± 0.01%/day) and P1 (1.99 ± 0.01%/day), with significant differences among treatments (p < 0.05). Likewise, the lowest FCR was observed in P2 (1.23 ± 0.04), followed by P3 (1.30 ± 0.02), whereas P1 exhibited the highest FCR value (1.48 ± 0.12). Although the FCR values of P2 and P3 did not differ significantly (p > 0.05), both treatments showed significantly lower FCR values than the control treatment (p < 0.05).

The highest FE was observed in P2 (81.22 ± 3.26%), followed by P3 (76.44 ± 1.42%) and P1 (67.94 ± 5.34%). The FE values in P2 and P3 were not significantly different (p > 0.05); however, both were significantly greater than that of P1 (p < 0.05).

The SR did not differ significantly among treatments (p > 0.05). Fish receiving LAB NJ19 exhibited the highest SR (77.50 ± 12.58%), followed by fish receiving EM-4 (72.50 ± 9.57%) and the control group (67.50 ± 9.57%).

### Effects of LAB NJ19 on water quality

Water quality measurements recorded throughout the experimental period are presented in Table 2 [30, 31]. Temperature and pH remained relatively stable across all treatments and were within acceptable ranges for common carp culture. Water temperature ranged from 27.3 ± 0.09°C in P1 to 27.55 ± 0.11°C in P2, whereas pH values ranged from 6.80 ± 0.04 to 7.09 ± 0.05.

**Table 2:** Water quality parameters monitored during the 40-day rearing period of common carp (*C. carpio*) fed control diet (P1), LAB NJ19-supplemented diet (P2), or commercial probiotic EM-4-supplemented diet (P3). Values represent mean ± standard deviation of daily measurements.

Treatment	Temperature (°C)	pH	DO (mg/L)
P1 (Control)	27.30 ± 0.09^a^	6.80 ± 0.04^a^	4.21 ± 0.20^a^
P2 (LAB probiotic NJ19)	27.55 ± 0.11^b^	7.01 ± 0.04^b^	5.28 ± 0.16^b^
P3 (Commercial probiotic EM-4)	27.52 ± 0.11^b^	7.09 ± 0.05^c^	6.76 ± 0.22^c^
Standard value	25.2–32.2°C [30]	6.5–9.0 [31]	> 5.0 mg/L [30]

Values are presented as mean ± standard deviation. Different superscript letters within a column indicate significant differences among treatments (p < 0.05).

DO differed significantly among treatments (p < 0.05). The highest DO concentration was recorded in P3 (6.76 ± 0.22 mg/L), followed by P2 (5.28 ± 0.16 mg/L), whereas the lowest value was observed in P1 (4.21 ± 0.20 mg/L). These results indicate that probiotic supplementation improved DO conditions compared with the control treatment. Overall, the observed water quality parameters remained within acceptable limits for common carp culture.

### Proximate composition of the basal diet

The proximate composition of the basal diet, PF-1000 (MS Prima Feed, Surabaya, Indonesia), consisted of 39%–41% crude protein, ≥5% crude lipid, ≤6% crude fiber, ≤16% ash, and ≤10% moisture, according to the manufacturer’s specifications.

## DISCUSSION

### Identification and probiotic characteristics of LAB NJ19

Molecular identification of probiotic LAB NJ19 further confirms its probiotic potential. Based on *16S rRNA* gene sequencing, probiotic LAB NJ19 showed high similarity to *E. faecium*, and phylogenetic analysis confirmed its clustering within the *E. faecium* clade. This phylogenetic analysis reinforces NJ19 classification as a probiotic LAB. This finding is consistent with previous studies that reported *E. faecium* is commonly isolated from the digestive tracts of fish. Similar findings have been reported that *E. faecium* was identified among LAB isolated from the intestinal tract of Nile tilapia [30] and from fish viscera [31]. Furthermore, host-associated LAB strains have been shown to exhibit greater colonization ability and probiotic effectiveness than non-indigenous strains [32].

The identification of LAB NJ19 as *E. faecium* is supported by previous studies, which reported that this species is widely regarded as a probiotic with antimicrobial and metabolic capabilities. *E. faecium* can produce antimicrobial compounds, such as bacteriocins, that inhibit pathogenic bacteria and enhance host health. Fish-derived *E. faecium* strains have been reported to inhibit the pathogenic bacteria, including *A. hydrophila*, and to improve host protection in common carp [32]. In addition, bacteriocin-producing *E. faecium* strains have been isolated from fish viscera[31]. Furthermore, genomic and functional studies have confirmed the presence of genes in *E. faecium* that encode bacteriocins and other bioactive compounds, thus supporting its role in inhibiting pathogens and functioning as a probiotic [33].

This research shows that probiotic LAB NJ19 (P2) notably improved fish growth performance, as demonstrated in Table 1. The probiotic LAB NJ19 treatment exhibited the highest final weight (8.48 ± 0.31 g), absolute weight gain (7.31 ± 0.29 g), SGR (2.13 ± 0.03% day^−1^), and FE (81.22 ± 3.26%) among the groups. In this study, the indigenous probiotic LAB NJ19 performed better than the commercial probiotic EM-4, indicating potential advantages of host-associated probiotics derived from phylogenetically related species. Findings from this study indicate that probiotic LAB NJ19 may improve growth and feed utilization efficiency in common carp. This finding is consistent with previous studies demonstrating that dietary supplementation with *E. faecium* significantly enhances growth performance, feed utilization efficiency, and survival in aquaculture species [34]. These results further support the notion that supplementation with *E. faecium* improves SGR FCR, feed conversion efficiency, digestive enzyme activity, and gut microbial balance [35].

### Effects of LAB NJ19 on growth performance and feed utilization

The improvement in growth performance may be associated with enhanced digestive processes. Several reports have shown that LAB produce extracellular enzymes that facilitate the breakdown of complex macromolecules into simpler compounds, enabling efficient nutrient absorption and utilization. Moreover, LAB help maintain the digestive tract’s microbial balance by suppressing pathogenic bacteria, thereby improving digestive efficiency and metabolic performance and promoting beneficial microbial communities. This study confirms that *E. faecium* strains have been reported to enhance digestive enzyme activity and gut microbiota balance, thereby directly improving nutrient absorption and metabolic efficiency in fish [36]. Therefore, modulation of gut microbiota may be one of the mechanisms through which *E. faecium* improves digestive efficiency and metabolic performance in fish.

Probiotic LAB NJ19 exhibited the highest amylase activity among LAB isolates, indicating its strong ability to hydrolyze dietary starch into simpler sugars such as maltose and glucose. This enzymatic activity is particularly important in omnivorous fish such as *C. carpio*, where carbohydrate digestion plays a major role in energy metabolism and growth. Enhanced amylase activity has been shown to increase carbohydrate utilization efficiency and energy availability, thereby improving growth performance and FE in fish [35]. *E. faecium*-based probiotic supplementation was associated with improved growth performance, FCR, feed conversion efficiency, digestive enzyme activity, and gut microbial balance.

The addition of LAB NJ19 to the digestive tract of fish improves feed digestibility by producing lactic acid, which lowers the pH and stimulates endogenous enzyme activity. These results are consistent with reports that administering probiotics improves nutrient digestibility, digestive enzyme activity, and growth performance. Moreover, probiotic *E. faecium* has been shown to improve FCR and FE by enhancing enzymatic digestion and microbial balance in the gut [35].

In addition, the effectiveness of probiotic activity is influenced by the nutritional composition of the basal feed. The proximate composition of the PF-1000 feed, with high protein (39%–41%) and sufficient energy, supports growth and metabolic processes. Carbohydrates, which are not listed directly, are present as a nitrogen-free extract and serve as an important energy source. Therefore, the high amylase activity of probiotic LAB NJ19 is highly relevant to enhancing carbohydrate digestion, indicating a beneficial association between feed composition and probiotic enzymatic activity.

EM-4 contains LAB (*Lactobacillus* sp.), photosynthetic bacteria (*Rhodopseudomonas* sp.), and yeast (*Saccharomyces* sp.), which contribute to digestive processes and nutrient availability. However, despite its multistrain composition, EM-4 showed lower performance compared with LAB NJ19, suggesting that a functionally dominant strain with high enzymatic activity may be more effective than mixed microbial cultures with lower specific activity. It also shows NJ19 indicates a better adaptation to fish intestinal conditions, which may be more effective than mixed microbial cultures with broader but less targeted functional activity. an intestinal *E. faecium* strain with probiotic traits, including tolerance to gastrointestinal stress conditions and inhibition of pathogenic bacteria [37].

**Figure 5:**
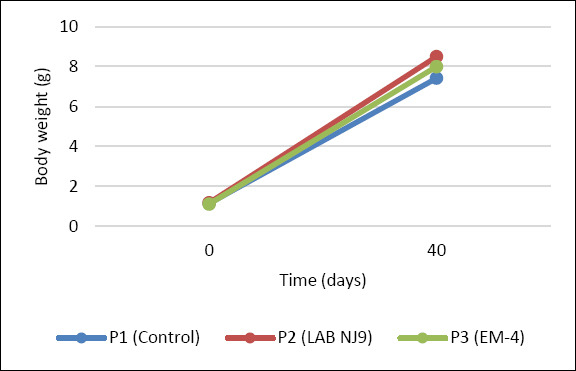
Growth curve of common carp (*C. carpio*) based on body weight during a 40-day feeding trial. P1 = Control, P2 = LAB NJ19, and P3 = commercial probiotic (EM-4). Values represent mean body weight.

**Figure 6:**
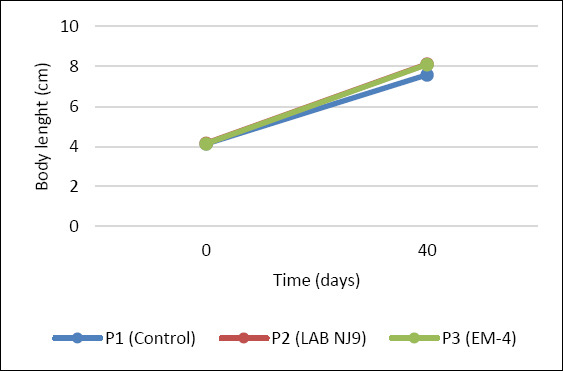
Growth curve of common carp (*C. carpio*) based on body length during a 40-day feeding trial. P1 = Control, P2 = LAB NJ19, and P3 = commercial probiotic (EM-4). Values represent mean body length.

This study demonstrated that adding probiotics affected the SGR and FCR of common carp. The highest SGR was obtained with LAB NJ19, followed by EM-4, whereas the control group had the lowest value. Feed supplemented with LAB NJ19 and EM-4 resulted in lower FCR values, indicating more efficient feed utilization. The observed correlation between increased SGR and decreased FCR suggests that the nutrients in the feed were utilized more effectively for growth than for the metabolism of waste products (Figures 5 and 6). The results are consistent with previous reports that probiotic *E. faecium* improves feed conversion efficiency and promotes nutrient assimilation in fish [34, 35]. *E. faecium* supplementation can improve growth performance, feed conversion efficiency, digestive function, and gut microbial balance in fish.

The addition of the probiotic LAB NJ19 and EM-4 improved FE and SR, although SR did not differ significantly among treatments. The variation observed in SR may be due to handling stress, water exchange, and initial fish conditions, which are common factors in experimental systems. In contrast, some studies suggest that certain handling practices, when optimized, may not significantly impact SR, indicating that stress management strategies can mitigate adverse effects.

Probiotic LAB NJ19 and EM-4 supplements improve the balance of intestinal microflora, enhance immune responses, and increase resistance to stress and infection. This finding broadly supports the work of other studies that *E. faecium* enhances immune responses and significantly increases resistance to pathogenic infections, particularly those caused by *A. hydrophila*, thereby improving fish survival [32, 34].

### Effects of LAB NJ19 on water quality

The water quality parameters were all within acceptable ranges across all treatments (Table 2). Temperature and pH levels remained stable for common carp culture. However, DO differed among treatments, with higher values observed in the probiotic-supplemented groups than in the control. The highest DO concentration was recorded in the EM-4 treatment, followed by LAB NJ19, whereas the lowest value was observed in the control treatment. Higher DO availability is important for aerobic metabolism, energy production, and physiological performance in fish.

The higher DO values observed in the probiotic-treated groups may be associated with improved microbial balance and more efficient organic matter decomposition in the culture system. EM-4 contains mixed microorganisms, including LAB, photosynthetic bacteria, and yeast, which may contribute to organic matter degradation and explain its higher DO value compared with LAB NJ19. Meanwhile, LAB NJ19 maintained DO above the recommended level and simultaneously produced the best growth performance and feed utilization efficiency. The potential role of probiotics in improving microbial balance in aquaculture systems. *E. faecium* can be administered through feed and/or water and can modulate fish-associated microbial communities [34, 36].

However, the mechanism underlying these differences remains unclear, as ammonia, nitrite, and microbial community structure were not measured. Therefore, the effects of probiotics on water quality should be interpreted with caution.

### Implications for aquaculture and future research

In summary, the enhanced performance associated with probiotic LAB NJ19 may be attributed to its high enzymatic activity, potential ability to improve nutrient digestion, capacity to maintain microbial balance, and contribution to environmental stability. These findings emphasize the importance of selecting probiotics based on their functional characteristics rather than their taxonomic identity alone.

This study presents novel evidence that the indigenous *E. faecium* isolate can serve as an effective alternative to commercial probiotics in aquaculture applications. Further studies are needed to determine the mechanisms underlying the probiotic effects of LAB NJ19. This should be achieved by directly evaluating digestive enzyme activity, gut microbiota composition, and immune responses. Furthermore, we recommend conducting comprehensive assessments of water quality parameters, including ammonia, nitrite, and nitrate, and extending the experimental period to validate the stability and functional performance of the probiotic LAB NJ19 in aquaculture systems.

## CONCLUSION

Dietary supplementation with the host-associated probiotic LAB NJ19 significantly improved the growth performance and feed utilization of common carp (*C. carpio*). Fish receiving LAB NJ19 exhibited the highest absolute weight gain (7.31 ± 0.29 g), SGR (2.13 ± 0.03%/day), and FE (81.22 ± 3.26%), together with the lowest FCR (1.23 ± 0.04), indicating superior nutrient utilization compared with both the control and commercial probiotic treatments. In addition, probiotic supplementation helped maintain acceptable water quality, with higher DO values observed in the probiotic-treated groups than in the control group. Although the highest DO value was recorded in the commercial probiotic treatment, LAB NJ19 produced the best growth performance and feed utilization efficiency. Although SR was numerically higher in the LAB NJ19 group, no significant differences were detected among treatments.

The beneficial effects of LAB NJ19 are likely attributable to its host-associated origin, high amylase activity, ability to enhance nutrient digestion, and capacity to maintain microbial balance in the digestive tract.. These findings demonstrate that a probiotic isolated from a phylogenetically related fish species can provide measurable benefits for aquaculture production and may offer advantages over conventional commercial probiotics.

A major strength of this study is the evaluation of an indigenous LAB isolate obtained from the digestive tract of Naleh fish (*Barbonymus* sp.), providing novel evidence for the application of host-associated probiotics in common carp culture. However, several limitations should be acknowledged. The study did not directly evaluate digestive enzyme activity, intestinal microbiota composition, immune responses, or key water quality indicators such as ammonia, nitrite, and nitrate. In addition, the experimental period was limited to 40 days, and the viable bacterial count in the supplemented feed was not quantified.

Future research should investigate the mechanisms underlying the probiotic effects of LAB NJ19 through detailed analyses of digestive physiology, gut microbiota, immune responses, and nutrient metabolism. Long-term feeding trials and comprehensive water quality assessments are also needed to validate the stability, safety, and effectiveness of LAB NJ19 under commercial aquaculture conditions.

Overall, LAB NJ19 demonstrated considerable potential as a sustainable host-associated probiotic for common carp culture. The use of indigenous *E. faecium* strains such as LAB NJ19 may contribute to improved fish productivity, enhanced feed utilization, and better environmental management, thereby supporting the development of more efficient and sustainable freshwater aquaculture systems.

## DATA AVAILABILITY

The supplementary data can be made available from the corresponding author upon request.

## AUTHORS’ CONTRIBUTIONS

CND, PNA, and CM: Planned the study, conducted the screening process, performed data analysis, and drafted the manuscript. CY and YSI: Performed data analysis and interpreted the results. DY, ZAM, and SS: Interpreted the results and revised the manuscript. All authors have read and approved the final manuscript.
